# Interpregnancy interval and adverse perinatal outcomes: A within‐individual comparative method

**DOI:** 10.1002/hsr2.2313

**Published:** 2024-08-19

**Authors:** Maria Sevoyan, Marco Geraci, Edward A. Frongillo, Jihong Liu, Nansi S. Boghossian

**Affiliations:** ^1^ Department of Epidemiology and Biostatistics Arnold School of Public Health, University of South Carolina Columbia South Carolina USA; ^2^ MEMOTEF Department, School of Economics Sapienza University of Rome Roma Italy; ^3^ Department of Health Promotion, Education, and Behavior Arnold School of Public Health, University of South Carolina Columbia South Carolina USA

**Keywords:** adverse perinatal outcomes, birth spacing, interpregnancy interval, sibling comparison, within‐individual comparison, within‐woman comparison

## Abstract

**Background and Aim:**

Previously observed associations between interpregnancy interval (IPI) and perinatal outcomes using a between‐individual method may be confounded by unmeasured maternal factors. This study aims to examine the association between IPI and adverse perinatal outcomes using within‐individual comparative analyses.

**Methods:**

We studied 10,647 individuals from the National Institute of Child Health and Human Development Consecutive Pregnancies Study in Utah with ≥3 liveborn singleton pregnancies. We matched two IPIs per individual and used conditional logistic regression to examine the association between IPI and adverse perinatal outcomes, including preterm birth (PTB, <37 weeks’ gestation), small‐for‐gestational‐age (SGA, <10th percentile of sex‐specific birthweight for gestational age), low birthweight (LBW, <2,500 g), and neonatal intensive care unit (NICU) admission. Point and 95% confidence interval (CI) estimates were adjusted for factors that vary across pregnancies within individuals.

**Results:**

CIs did not unequivocally support either an increase or a decrease in the odds of PTB (adjusted odds ratio [aOR]: 1.31, 95% CI: 0.87, 1.96), SGA (aOR: 0.81, 95% CI: 0.51, 1.28), LBW (aOR: 1.59, 95% CI: 0.90, 2.80), or NICU admission (aOR: 0.96, 95% CI: 0.66, 1.40) for an IPI <6 months compared to 18–23‐months IPI (reference), and neither did the CIs for the aOR of IPIs of 6–11 and 12–18 months compared to the reference. In contrast, an IPI ≥24 months was associated with increased odds of LBW (aOR: 1.66, 95% CI: 1.03, 2.66 for 24–29 months; aOR: 2.27, 95% CI: 1.21, 4.29 for 30–35 months; and aOR: 2.09, 95% CI: 1.17, 3.72 for ≥36 months).

**Conclusions:**

Using a within‐individual comparative method, we did not find evidence that a short IPI compared to the recommended IPI of 18–23 months was associated with increased odds of PTB, SGA, LBW, and NICU admission. IPI ≥ 24 months was associated with increased odds of delivering an LBW infant.

## Key points

What is already known

- Short and long interpregnancy intervals (IPIs) were associated with increased odds of adverse perinatal outcomes using a between‐individual method.

What this study adds

- The within‐individual comparative method showed that a short compared to recommended IPI was not associated with increased odds of small‐for‐gestational‐age, preterm birth, low birthweight (LBW), and neonatal intensive care unit admission. IPI ≥ 24 months was associated with increased odds of delivering an LBW newborn.

Clinical implications

- Previously observed associations between IPI and adverse perinatal outcomes using a between‐individual method may be due to unmeasured confounding. Intervening on a short IPI itself may not be beneficial for decreasing the prevalence of adverse perinatal outcomes.

## 1 INTRODUCTION

Low birthweight (LBW), small‐for‐gestational‐age (SGA), and preterm birth (PTB) are associated with increased risk of perinatal mortality and morbidity as well as long‐term developmental outcomes.^1^, ^2^, ^3^ Nonoptimal birth spacing between successive pregnancies may contribute to the risk of these outcomes.^4^, ^5^, ^6^

Studies from different geographic regions consistently show that short and long interpregnancy intervals (IPIs) are associated with an increased risk of subsequent adverse perinatal outcomes, such as PTB, SGA, and LBW.^4^, ^5^, ^6^ The dose–response relationship between the adverse perinatal outcomes and IPI is frequently described in the literature as J‐shaped, that is, the proportion of adverse outcomes is higher for IPI < 6 and ≥60 months but is lower for the interval between 18 and 23 months.^5^, ^7^ According to the World Health Organization, an optimal IPI of at least 24 months is recommended after a live birth given the lowest risk of adverse perinatal outcomes.^8^ This recommendation was based on studies mostly from low‐ and middle‐income countries, and thus its relevance to individuals from high‐income countries such as the United States remains unclear.^6^

Most studies examining the association between IPI and perinatal outcomes have used a between‐individual comparative method, where the perinatal outcomes of the individuals with either short or long IPIs were compared to the outcomes of other individuals with the recommended IPI. The reported positive associations of short and long IPIs with adverse perinatal outcomes, however, might be attributed to unobserved confounding.^9^, ^10^ To overcome potential confounding issues, recent studies examined the relationship between IPI and adverse perinatal outcomes using a within‐individual comparative method, whereby each study participant serves as their own control. That is, the contrast between two perinatal outcomes, one observed after a nonoptimal interval and one after the optimal or recommended IPI, is carried out for the same pregnant individual who provides data for at least two intervals (e.g., three successive deliveries). In those studies, the estimates obtained from the within‐individual comparative method were mostly attenuated as compared to those based on the between‐individual method,^11^, ^12^, ^13^, ^14^ thus supporting the speculation that previously observed associations between IPI and perinatal outcomes might be partly explained by individual heterogeneity.^15^

Although the within‐individual comparative method has recently been utilized in IPI research, such studies are relatively limited in the United States.^14^ Most existing within‐individual studies in this area often lack information on key time‐varying confounders, including socioeconomic factors, prepregnancy body mass index (BMI), smoking during pregnancy, and maternal co‐morbidities.

Experts in the field have highlighted the need to adjust for confounders that vary between individual's pregnancies. They also emphasize the importance of assessing the generalizability of the cohort used in the within‐individual analysis and exploring the potential influence of the outcomes of the previous birth and pregnancy losses on the IPI and perinatal outcomes relationship by conducting additional sensitivity analysis.^16^

Given the limited studies conducted in the United States using a within‐individual comparative method,^14^ the limitations of the existing studies and the recent recommendations on good practices in IPI studies,^16^ we aimed to examine the association between IPI and PTB, SGA, LBW, and neonatal intensive care unit (NICU) admission in the subsequent pregnancy using a within‐individual comparative method in a cohort of US individuals from the state of Utah.

## 2 METHODS

### 2.1 Data source

We conducted a retrospective matched cohort study to examine the association between IPI and adverse perinatal outcomes among individuals from the *Eunice Kennedy Shriver* National Institute of Child Health and Human Development (NICHD) Consecutive Pregnancies Study.^17^ Data on consecutive pregnancies were collected from 51,086 individuals delivering at ≥20 weeks of gestation in 20 hospitals in the state of Utah during the period of 2002–2010. During the study period, around 78.2% (*n* = 39,974) of the individuals had only two deliveries and 21.8% of individuals (*n* = 11,112) contributed three or more deliveries. Detailed information on maternal demographics, past medical history, reproductive and perinatal history, pregnancy, labor and delivery outcomes, postpartum, and newborns was extracted from electronic medical records and in‐patient hospital discharge summaries.^17^ Codes from the International Classification of Diseases, ninth revision (ICD‐9) from maternal and newborn discharge summaries were linked to each delivery. All participating institutions obtained approval for the study and a waiver of informed consent from their individual institutional review boards.

### 2.2 Study population

**Figure 1:**
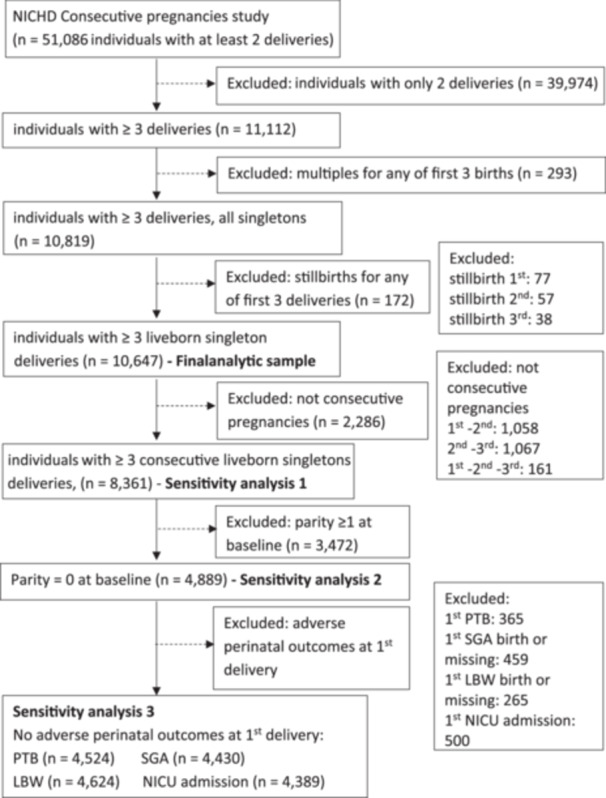
Flow chart of individuals included in the main and sensitivity analyses, National Institute of Child Health and Human Development (NICHD) consecutive pregnancies study, Utah, 2002–2010. A total of 10,647 individuals out of a total of 51,086 individuals from the NICHD consecutive pregnancies study met the inclusion criteria for this study (individuals with ≥ 3 liveborn singleton deliveries during the study period). LBW, low birthweight; NICU, neonatal intensive care unit; PTB, preterm birth; SGA, small‐for‐gestational‐age.

Of the total of 51,086 individuals enrolled in the NICHD Consecutive Pregnancies Study, we excluded individuals with only two births during the study period as these would have contributed to only one IPI. We also excluded individuals with multiple gestations or stillbirths for any of the first three pregnancies during the study period (Figure 1). Multiple gestations were excluded due to their unique physiological impact and higher nutritional demands compared to singleton pregnancies, the potential for confounding due to factors such as assisted reproductive technologies and secondary infertility, and the limited sample size available for stratified analysis. We excluded stillbirths to minimize potential confounding, as individuals experiencing stillbirth often have shorter IPIs and underlying conditions that can affect subsequent pregnancies.^18^ The final analytical sample included 10,647 individuals with ≥3 liveborn singleton deliveries during the study period.

### 2.3 Exposure

The exposure, IPI, was defined as the time interval between the previous live birth and the estimated conception date of the subsequent pregnancy, which was calculated as the last menstrual period for the subsequent pregnancy minus the date of the previous delivery. IPI was grouped into seven categories: 0–5, 6–11, 12–17, 18–23 (reference), 24–29, 30–35, and ≥36 months. For each study participant, there were two IPIs: interval between the first delivery and the second pregnancy's conception date and the second interval between the second delivery and the third pregnancy's conception date. If a study participant had more than three deliveries (i.e., more than two IPIs) during the study period, only the first three deliveries (and first two IPIs) were examined.

### 2.4 Outcomes

We studied four binary perinatal outcomes: PTB (delivery at <37 completed weeks of gestation), SGA (<10th percentile of national birthweight centiles for sex and gestational age),^19^ LBW (birthweight <2,500 g), and NICU admission. Gestational age was based on the best obstetric estimate obtained from electronic medical records.

### 2.5 Covariates

In our study's method, each pregnant individual represents a separate stratum, thus individual‐specific factors (measured and unmeasured) that are shared between pregnancies can be controlled for by appropriate regression modeling.^20^ The model, however, needs to also adjust for factors that change across an individual's pregnancies. Thus, we adjusted for the variables that can potentially change between pregnancies within the same individual, including maternal age at the time of each delivery, insurance type, marital status, smoking during pregnancy, prepregnancy BMI, parity, mode of delivery, and maternal co‐morbidity status. Maternal co‐morbidity status included the presence of at least one of the following: heart disease, chronic hypertension, diabetes mellitus, thyroid disorder, asthma, and renal disease. Maternal chronic conditions were ascertained from medical records and supplemented with ICD‐9 codes. These potential confounders were selected based on previous literature^5^, ^16^, ^21^, ^22^ and availability.

### 2.6 Statistical analysis

Conditional logistic regression was used to perform within‐individual comparisons.^23^ In this regression, only individuals with discordant outcomes contribute to the overall estimate of the effect.^20^ The unadjusted and adjusted odds ratios (ORs) and 95% confidence intervals (CI) for the association between IPI and each of the adverse perinatal outcomes were estimated. All analyses were performed using SAS® 9.4 software.

#### 2.6.1 Missing data

We performed a complete case analysis. Since the proportions of missing data for incomplete variables were small (ranging from 0.019% for marital status to 2.7% for prepregnancy BMI), the application of missing data methods (e.g., imputation) was not deemed necessary.

#### 2.6.2 Sensitivity analyses

We performed sensitivity analyses to examine the robustness of the estimates with respect to three different data selection criteria. (1) Scenario A: we restricted our sample to only individuals with consecutive first, second, and third deliveries (*n* = 8,361) during the study period, given the possible influence of pregnancy losses on the association between IPI and outcomes.^16^ We examined whether deliveries were consecutive by checking if gravidity increased more than parity, indicating that a study participant had a pregnancy loss before 20 weeks of gestation. (2) Scenario B: we further restricted the Scenario A sample to individuals who were nulliparous at the beginning of the study (*n* = 4,889). (3) Scenario C: we further restricted the Scenario B sample to individuals without corresponding adverse perinatal outcomes in the first delivery (Figure 1).

#### 2.6.3 Supplementary analyses

We performed several supplementary analyses to explore the association between IPI and outcomes in more depth. (1) Given that individuals with ≥3 births may be different from individuals with only 2 births, we assessed the generalizability of our findings by comparing the characteristics and perinatal outcomes of these two groups of individuals. We also performed between‐individual comparisons (unconditional logistic regression) for the sample of individuals with only two births and estimated the adjusted ORs (aORs) and 95% CIs for the association between IPI and adverse outcomes. (2) We used post‐birth intervals^9^, ^13^ in a negative controls^24^ analysis to detect the potential influence of unmeasured confounding on the association between post‐birth IPI (IPI between second and third birth) and odds of perinatal outcomes of the second birth for our sample of individuals with ≥3 births. The goal was not to investigate a causal relationship since, due to the chronological sequence of the events, the post‐birth interval cannot have an effect on the outcome of the second birth. Rather, the presence of an association may reflect the impact of factors that are common to both adverse perinatal outcomes and the length of the IPI.

## 3 RESULTS

The final analytical sample consisted of 10,647 individuals, which accounted for 21,294 IPI observations (two per study participant). At the second birth, around 58% of individuals conceived after IPIs of <18 months (6.7% after IPI <6 months), and around 20% conceived after IPIs ≥24 months. At the third birth, around 47% of individuals conceived after IPIs <18 months (4.7% after IPI < 6 months), and around 31% of deliveries occurred after IPIs ≥24 months. Only about 22% of individuals conceived after the recommended IPI of 18–23 months after their first and second deliveries. Most individuals were white (89%), under 25 years, married (89%), with private insurance (75%), and with normal prepregnancy BMI (63%) at the study entry (Table 1).

**Table 1:** Maternal and newborn characteristics of the sample (*n* = 10,647), NICHD consecutive pregnancies study, Utah, 2002–2010.

	First birth, *n* (%)	Second birth, *n* (%)	Third birth, *n* (%)
*Pregnant individual characteristics*			
IPI, months, mean (SD)	n/a	17.1 (8.8)	19.6 (9.8)
IPI, months	n/a		
0–5		715 (6.7)	500 (4.7)
6–11		2247 (21.1)	1719 (16.2)
12–17		3157 (29.7)	2760 (25.9)
18–23		2343 (22.0)	2351 (22.1)
24–29		1223 (11.5)	1715 (16.1)
30–35		561 (5.3)	887 (8.3)
≥36		401 (3.8)	715 (6.7)
Race			
White	9409 (88.5)		
Non‐White	1226 (11.5)		
Age at birth, years, mean (SD)	24.7 (3.9)	26.8 (4.0)	29.2 (4.1)
Age at birth, years			
14–19	814 (7.7)	279 (2.6)	57 (0.5)
20–24	4656 (43.7)	2827 (26.6)	1221 (11.5)
25–29	3980 (37.4)	4998 (46.9)	4556 (42.8)
30–34	1043 (9.8)	2130 (20.0)	3743 (35.2)
≥35	154 (1.5)	413 (3.9)	1070 (10.1)
Insurance			
Private	7927 (74.5)	7976 (74.9)	7984 (75.0)
Public	2720 (25.6)	2671 (25.1)	2663 (25.0)
Marital status			
Not married	1152 (10.8)	853 (8.1)	760 (7.1)
Married	9494 (89.2)	9792 (92.0)	9887 (92.9)
Prepregnancy BMI, kg/m^2^			
Underweight (<18.5)	701 (6.7)	597 (5.7)	490 (4.7)
Normal (18.5–25)	6590 (63.0)	6107 (58.3)	5829 (55.4)
Overweight (25–30)	2031 (19.4)	2278 (21.7)	2380 (22.6)
Obese (≥30)	1137 (10.9)	1500 (14.3)	1822 (17.3)
Parity			
0	6154 (57.8)	0	0
1	2643 (24.8)	6154 (57.8)	0
2	1106 (10.4)	2643 (24.8)	6154 (57.8)
≥3	744 (7.0)	1850 (17.4)	4493 (42.2)
Smoking during pregnancy			
No	10,481 (98.5)	10,387 (97.7)	10,367 (97.5)
Yes	156 (1.5)	247 (2.3)	268 (2.5)
Alcohol use during pregnancy			
No	10,538 (99.2)	10,542 (99.2)	10,526 (99.1)
Yes	87 (0.8)	85 (0.8)	98 (0.9)
Pre‐existing diabetes mellitus	101 (1.0)	175 (1.6)	235 (2.2)
Pre‐existing hypertension	37 (0.4)	71 (0.7)	101 (1.0)
Chronic conditions^a^	1190 (11.2)	1632 (15.3)	2017 (18.9)
Cesarean section	1336 (12.6)	1459 (13.7)	1728 (16.2)
*Newborn characteristics*			
Female	5146 (48.3)	5069 (47.6)	5169 (48.6)
Birthweight (g), mean (SD)	3339.0 (508.5)	3361.0 (484.8)	3367.1 (495.8)
PTB	762 (7.2)	737 (6.9)	816 (7.7)
SGA	833 (7.8)	557 (5.2)	552 (5.2)
LBW	462 (4.3)	367 (3.5)	387 (3.6)
NICU admission	930 (8.7)	724 (6.8)	860 (8.1)

*Note*: Percentages are column percentages.

Abbreviations: BMI, body mass index; IPI, interpregnancy interval; LBW, low birthweight; n/a, not applicable; NICU, neonatal intensive care unit; PTB, preterm birth; SD, standard deviation; SGA, small‐for‐gestational‐age.

^a^ Individual's chronic conditions include having at least one of the following conditions: heart disease, chronic hypertension, diabetes mellitus, thyroid disorder, asthma, renal disease.

Missing: race (*n* = 12); marital status (*n* = 1), prepregnancy BMI (*n* = 188), maternal smoking (*n* = 10), alcohol use during pregnancy (*n* = 22), birthweight (*n* = 4), SGA (*n* = 12), LBW (*n* = 4) at the first birth; marital status (*n* = 2), BMI (*n* = 165), maternal smoking (*n* = 13), alcohol use during pregnancy (*n* = 20), birthweight (*n* = 7), SGA (*n* = 14), LBW (*n* = 7) at the second birth; BMI (*n* = 126), maternal smoking (*n* = 12), alcohol use during pregnancy (*n* = 23), birthweight (*n* = 2), SGA (*n* = 5), LBW (*n* = 2) at the third birth.

**Figure 2:**
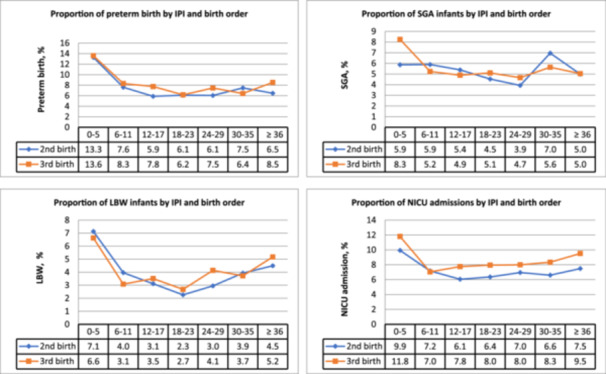
Proportion of adverse perinatal outcomes for second and third births by interpregnancy interval in individuals with ≥3 liveborn singleton pregnancies (*n* = 10,647), National Institute of Child Health and Human Development (NICHD) consecutive pregnancies study, Utah, 2002–2010. IPI, interpregnancy interval; LBW, low birthweight; NICU, neonatal intensive care unit; SGA, small‐for‐gestational‐age.

The unadjusted proportions of the perinatal outcomes during the second and third births are presented in Figure 2. The observed proportions of adverse perinatal outcomes in the sample were low. The distribution of these outcomes varied across different IPI categories, with a tendency for increased proportions in the shorter and longer IPI categories, compared to the recommended IPI of 18–23 months. The pattern of adverse outcomes by IPI categories was consistent for second and third births (Figure 2).

### 3.1 IPI and odds of adverse perinatal outcomes (within‐individual comparison)

The analysis did not demonstrate increased or decreased odds for any of the four perinatal outcomes associated with IPI intervals <18 months compared to IPI of 18–23 months (Table 2). For PTB, the largest OR was 1.31 (95% CI: 0.87, 1.96) and for LBW it was 1.59 (95% CI: 0.90, 2.80) for the shortest interval of <6 months, although these CIs include 1, indicating no definite increase or decrease in risk. Regarding SGA and NICU admission, the largest ORs of 1.12 (95% CI: 0.81, 1.55) and 1.15 (95% CI: 0.89, 1.48) were observed for the intervals of 6–11 and 12–17 months, respectively, though these estimates do not indicate a definite increase or decrease in risk.

**Table 2:** ORs and 95% CI for the association between interpregnancy interval and adverse perinatal outcomes in individuals with at least three liveborn singleton births (*n* = 10,647), NICHD consecutive pregnancies study, Utah, 2002–2010.

IPI, months	Unadjusted OR (95% CI)	Adjusted^a^ OR (95% CI)	IPI, months	Unadjusted OR (95% CI)	Adjusted^a^ OR (95% CI)
PTB			LBW		
0–5	1.17 (0.80, 1.71)	1.31 (0.87, 1.96)	0–5	1.36 (0.82, 2.25)	1.59 (0.90, 2.80)
6–11	0.86 (0.65, 1.15)	0.89 (0.65, 1.21)	6–11	1.17 (0.77, 1.77)	1.31 (0.82, 2.10)
12–17	1.09 (0.84, 1.41)	1.01 (0.76, 1.34)	12–17	1.37 (0.94, 2.00)	1.35 (0.89, 2.04)
18–23	1.00 (Reference)	1.00 (Reference)	18–23	1.00 (Reference)	1.00 (Reference)
24–29	1.14 (0.83, 1.59)	1.04 (0.73, 1.48)	24–29	**1.56** (**1.02, 2.39)**	**1.66** (**1.03, 2.66)**
30–35	1.08 (0.72, 1.61)	0.98 (0.64, 1.51)	30–35	**2.10** (**1.19, 3.72)**	**2.27** (**1.21, 4.29)**
≥36	1.01 (0.68, 1.49)	1.01 (0.66, 1.54)	≥36	**1.69** (**1.02, 2.80)**	**2.09** (**1.17, 3.72)**
SGA			NICU admission		
0–5	0.88 (0.58, 1.35)	0.81 (0.51, 1.28)	0–5	0.91 (0.65, 1.28)	0.96 (0.66, 1.40)
6–11	1.11 (0.81, 1.51)	1.12 (0.81, 1.55)	6–11	0.84 (0.66, 1.08)	0.89 (0.68, 1.16)
12–17	0.96 (0.72, 1.28)	1.00 (0.74, 1.34)	12–17	1.07 (0.84, 1.36)	1.15 (0.89, 1.48)
18–23	1.00 (Reference)	1.00 (Reference)	18–23	1.00 (Reference)	1.00 (Reference)
24–29	0.79 (0.56, 1.11)	0.79 (0.55, 1.14)	24–29	1.03 (0.79, 1.35)	1.07 (0.80, 1.42)
30–35	**1.54** (**1.00, 2.35)**	**1.73** (**1.11, 2.72)**	30–35	1.04 (0.75, 1.46)	1.00 (0.70, 1.43)
≥36	1.17 (0.72, 1.89)	1.21 (0.73, 2.01)	≥36	1.13 (0.80, 1.61)	1.13 (0.77, 1.65)

*Note*: Bold indicates statistical significance at the 5% level.

For unadjusted models, of the 10,647 pairs, 1,001 were informative for PTB, 881 for SGA, 552 for LBW, and 1,262 for NICU admission.

For adjusted models, 948 pairs were informative for PTB, 850 for SGA, 527 for LBW, and 1,222 for NICU admission (due to missing values for the covariates).

Abbreviations: CI, confidence interval; IPI, interpregnancy interval; LBW, low birth weight; NICU, neonatal intensive care unit; OR, odds ratio; PTB, preterm birth; SGA, small‐for‐gestational‐age.

^a^ Adjusted model accounted for characteristics that varied or could potentially vary between pregnancies within the same individual: individual's age, marital status, health insurance, prepregnancy BMI, chronic conditions, smoking during pregnancy, parity, mode of delivery; pregnancy order included in the adjusted model to allow for different intercepts at the second and third births.

Individual's chronic conditions include having at least one of heart disease, chronic hypertension, diabetes mellitus, thyroid disorder, asthma, and renal disease.

Intervals longer than 24 months were not associated with higher or lower odds of PTB or NICU admission. The highest OR of 1.04 (95% CI: 0.73, 1.48) for PTB and OR of 1.13 (95% CI: 0.77, 1.65) for NICU admission were observed for IPI 24–29 and ≥36 months, respectively. Since these CIs include 1, however, these results do not definitely indicate an increase or decrease in odds for these outcomes. In contrast, IPI longer than 24 months compared to the recommended IPI of 18–23 months was associated with increased odds of delivering an LBW infant. For LBW, the OR ranged from 1.66 (95% CI: 1.03, 2.66) for interval 24–29 months to OR of 2.27 (95% CI: 1.21, 4.29) for interval 30–35 months and OR of 2.09 (95% CI: 1.17, 3.72) for interval ≥36 months.

### 3.2 Sensitivity analyses

The results of the sensitivity analyses restricted to individuals with three consecutive births during the study period (Scenario A), further restricted to individuals with three consecutive births and nulliparous at study baseline (i.e., with parity 0, 1, 2) (Scenario B), and further restricted to the individuals without adverse perinatal outcomes at the first birth (Scenario C) were generally consistent with the results obtained from the main analyses (Supporting Information S1: Table S1). In Scenarios B and C, the adjusted estimates of the ORs for LBW were still greater than 1 at longer IPIs, although the associated larger uncertainty meant that most of the *p* values were greater than 0.05.

### 3.3 Supplementary analyses

#### 3.3.1 Between‐individual comparison

The supplementary analysis showed that individuals with only two births compared to those with ≥3 births on average waited longer after the first birth to conceive (mean IPI 22.4 vs. 17.1 months), were older at the second birth (mean age 28.4 vs. 26.8), were more likely to be obese or overweight at the beginning of their second pregnancy (41% vs. 36%), and more likely had comorbidities (18% vs. 15%) (Supporting Information S1: Table S2). The proportions of the adverse perinatal outcomes for individuals with only two births and ≥3 births were similar in both groups: higher rates of outcomes at short and long IPI intervals and lowest at the recommended IPI of 18–23 months. The adjusted results of the between‐individual comparisons (traditional logistic regression) for the sample of individuals with only two births showed that short IPI categories were associated with increased odds of PTB, LBW, and NICU admission. Longer IPIs were associated with increased odds of SGA, LBW, and NICU admission (Supporting Information S1: Table S3).

#### 3.3.2 Post‐birth interval

The results from the analysis using post‐birth intervals showed that a short post‐birth interval <6 months compared to the reference interval of 18‐23 months was associated with increased odds of PTB (aOR: 2.00, 95% CI: 1.41, 2.83), SGA (aOR: 1.62, 95% CI: 1.09, 2.42), and LBW (aOR: 2.52, 95% CI: 1.61, 3.92) in the previous pregnancy. In contrast, post‐birth intervals longer than 24 months were not associated with increased odds of any of the outcomes in the previous pregnancy (Supporting Information S1: Table S4).

## 4 DISCUSSION

### 4.1 Principal findings

We used a within‐individual comparative method design to examine the association between IPI and odds of PTB, LBW, SGA, and NICU admission in a sample of 10,647 individuals with ≥3 liveborn singleton deliveries from the state of Utah. Using a within‐individual comparative method, we did not find evidence that short IPI categories compared to the recommended IPI of 18–23 months were associated with increased odds of SGA, PTB, LBW, and NICU admission. However, longer IPIs compared to the reference IPI were associated with increased odds of delivering an LBW infant in the subsequent pregnancy. These results were similar in the sensitivity analyses.

Consistent with previous studies, traditional between‐individual comparative analyses for the sample of individuals with only two births demonstrated evidence of increased odds of adverse outcomes with both short and long IPI categories. We identified some differences in the characteristics of individuals with only 2 versus ≥3 births during the study period. This study showed that different methodologies (within‐individual and between‐individual comparisons) produce different results and may lead to different conclusions.

### 4.2 Strengths of the study

The within‐individual comparative method controls for all the characteristics that remain the same within the individual. In addition, detailed information available in our dataset for each delivery of the same individual provided the opportunity to further adjust for a range of factors that can potentially change across different pregnancies within the same study participant. Compared to other studies that utilized a within‐individual comparative method, we adjusted for several time‐varying characteristics (marital status, smoking during pregnancy, insurance type, maternal chronic co‐morbidities, pre‐pregnancy BMI) that may change across second and third pregnancies within the same individual. To overcome the common methodological issues with the birth spacing studies^10^ and following experts’ recommendations on good practices for within‐individual comparative method studies,^16^ we performed several sensitivity and supplementary analyses, including (a) an analysis to evaluate the potential influence of the pregnancy losses and outcome of the previous birth on IPI and perinatal outcomes relationship; (b) an analysis to assess the generalizability of the cohort used in the within‐individual analysis by comparing the results and characteristics of the cohorts of individuals with only 2 and ≥3 births; and (c) an analysis to adjust for the range of variables that vary between pregnancies, such as marital status, insurance type, smoking during pregnancy, pre‐pregnancy BMI, maternal comorbidities.

### 4.3 Limitations of the study

Most of our study population was White individuals, which represents a significant limitation in terms of racial diversity. Limited racial diversity may potentially impact the generalizability of our findings to more diverse and heterogeneous populations. Additionally, our study population consisted of low‐risk individuals, mostly composed of married individuals with private insurance, resulting in relatively low rates of adverse outcomes. Our estimates of the relationships of IPI with adverse outcomes may differ in other populations that are at high risk of adverse outcomes. Due to the study design, only individuals with ≥3 deliveries are included in our study population; as observed in our analysis, these individuals can be different from individuals with fewer deliveries.

### 4.4 Interpretation

Our study supports the recent findings that individual heterogeneity may be an explanation for the frequently observed associations between short IPI and adverse outcomes using a between‐individual comparison method. Traditional between‐individual comparisons for a sample of individuals with only two births demonstrated an increased risk associated with short and long IPI and adverse perinatal outcomes, which can indicate the inappropriate adjustment for confounders or presence of unmeasured confounders in between‐individual comparison studies. The post‐birth interval analysis results also demonstrated evidence of possible confounding by maternal factors. A strong positive association between short post‐birth interval and the risk of prior adverse perinatal outcomes may indicate confounding between short IPI and adverse outcomes. The absence of association between long post‐birth intervals and prior perinatal outcomes indicates that confounding may be less of an issue for the long IPI. Our post‐birth interval analysis results were similar to the results from other studies.^9^, ^13^

Compared to consistently demonstrated associations in previous research that used between‐individual comparisons, our study does not support a relationship between short IPI and adverse perinatal outcomes.^5^, ^6^ Our results of no evidence of increased odds of adverse perinatal outcomes after short IPI are mostly consistent with the previous research using within‐individual comparison.^11^, ^12^

As for the long IPIs, in a study of 38,000 individuals from Canada, Hanley et al. found that IPIs ≥60 months were associated with increased odds of LBW (aOR: 1.31, 95% CI: 1.02, 1.68) and NICU admission (aOR: 1.39, 95% CI: 1.02, 1.90).^12^ Class et al. in a study from Sweden demonstrated that IPI ≥ 60 months was associated with increased odds of SGA (aOR: 1.24, 95% CI: 1.10, 1.40), LBW (aOR: 1.24, 95% CI: 1.12, 1.38), and PTB (aOR: 1.17, 95% CI: 1.07, 1.27) in the sibling comparison models.^13^ In a study from Australia based on 40,000 individuals, there was evidence of increased odds of delivering an SGA infant after long IPI (aOR: 1.40, 95% CI: 1.11, 1.76 and aOR: 1.72, 95% CI: 1.04, 2.85 for IPI of 60–119 and ≥120 months, respectively) as compared to their siblings born after IPI of 18–23 months.^11^ In a study with more than five million individuals from Australia, Finland, Norway, and California, Tessema et al. consistently found increased odds of PTB and SGA following IPI categories ≥24 months.^25^ Despite using a within‐individual comparison method in a study with >300,000 individuals from California, however, Shachar et al. found a 20% increase in odds of PTB (95% CI: 1.13, 1.27) after IPI < 6 months compared to IPI of 18–23 months and no associations with long IPI.^14^ A Matched analysis in a study from China showed increased odds of PTB, LBW, and SGA after short IPIs and increased odds of PTB and LBW after IPI ≥ 36 months.^26^

The observed unfavorable association between longer intervals and LBW in our study might be explained by the “women's physiological regression” hypothesis proposed by Zhu et al.^7^ The hypothesis states that due to a long IPI between pregnancies (and individual aging), an individual's natural reproductive capacity gradually declines and becomes similar to a nulliparous individual. A systematic review, however, did not find evidence to support the “women's physiological regression” hypothesis as a biological mechanism explaining the effect of long IPIs on adverse perinatal outcomes.^27^

Increased odds of LBW after long IPI observed in our study may be a marker of biological or social mechanisms that lead individuals to have long IPI and also result in delivering an LBW infant. Conditions, such as subfertility and secondary infertility can contribute to a longer IPI. Studies suggest that subfertility and secondary infertility are associated with an increased risk of perinatal outcomes, irrespective of fertility treatment.^28^ Furthermore, individuals who undergo assisted reproductive technology treatments usually are older and have a higher risk of adverse pregnancy outcomes compared to those who conceive naturally.^29^ Not accounting for all the factors that change across pregnancies within the same individual may be the reason for the observed association between long IPI and increased odds of LBW.

## 5 CONCLUSION

Using a within‐individual comparison method, we did not find evidence that short IPIs compared to the reference IPI of 18–23 months were associated with increased odds of SGA, LBW, PTB, and NICU admission. Longer IPIs compared to the reference IPI of 18–23 months were associated with increased odds of delivering an LBW infant in the subsequent pregnancy. The previously observed consistent association between short IPI categories and perinatal outcomes may be due to residual confounding rather than the length of the IPI. Intervening on short IPI may not be beneficial for decreasing the rates of adverse perinatal outcomes.

## AUTHOR CONTRIBUTIONS

**Maria Sevoyan:** Conceptualization; data curation; formal analysis; methodology; software; validation; visualization; writing—original draft; writing—review and editing. **Marco Geraci:** Conceptualization; data curation; methodology; software; supervision; writing—review and editing. **Edward A. Frongillo:** Conceptualization; methodology; validation; visualization; writing—review and editing. **Jihong Liu:** Conceptualization; methodology; resources; writing—review and editing. **Nansi S. Boghossian:** Conceptualization; data curation; funding acquisition; methodology; project administration; resources; software; supervision; visualization; writing—original draft; writing—review and editing.

## CONFLICT OF INTEREST STATEMENT

The authors declare no conflict of interest.

## TRANSPARENCY STATEMENT

The lead author Nansi S. Boghossian affirms that this manuscript is an honest, accurate, and transparent account of the study being reported; that no important aspects of the study have been omitted; and that any discrepancies from the study as planned (and, if relevant, registered) have been explained.

## Supporting information

Supporting information.

## Data Availability

The data from this study are unable to be shared due to data use agreements with the data custodians.
