# Supplementary material for: Interpregnancy interval and adverse perinatal outcomes: A within‐individual comparative method
Source: Health Sci Rep. 2024 Aug 19;7(8):e2313. doi: 10.1002/hsr2.2313 (PMC11333538; doi:10.1002/hsr2.2313)
Supplement: Supplementary file 1 — Supporting information. [file HSR2-7-e2313-s001.docx]

**Supplementary tables**

**eTable 1.** Odds ratios (ORs) and 95% confidence intervals (CI) for the association between interpregnancy interval and adverse perinatal outcomes among individuals with three consecutive liveborn, singleton births during the study period (Scenario A); individuals with three consecutive liveborn, singleton births and nulliparous at baseline (parity 0, 1, 2) (Scenario B); individuals with three consecutive births and nulliparous at baseline (parity 0, 1, 2) excluding individuals with adverse perinatal outcome in the first delivery (Scenario C), NICHD consecutive pregnancies study, Utah, 2002-2010

|  | **Scenario A (N=8,361)** | | | **Scenario B (N=4,889)** | | | **Scenario C^a^** | | |
| --- | --- | --- | --- | --- | --- | --- | --- | --- | --- |
| **IPI, months** | **Unadjusted**  **OR (95% CI)** | **Adjusted ^b^**  **OR (95% CI)** | **Unadjusted**  **OR (95% CI)** | | **Adjusted ^b^**  **OR (95% CI)** | **Unadjusted**  **OR (95% CI)** | | **Adjusted ^b^**  **OR (95% CI)** |  |
| **PTB**^c^ |  |  |  | |  |  | |  |  |
| 0-5 | 1.24 (0.81, 1.89) | 1.37 (0.87, 2.17) | 1.14 (0.66, 1.98) | | 1.35 (0.74, 2.46) | 1.12 (0.60, 2.12) | | 1.40 (0.71, 2.76) |  |
| 6-11 | 0.93 (0.67, 1.29) | 0.94 (0.66, 1.35) | 0.82 (0.53, 1.25) | | 0.82 (0.51, 1.34) | 0.74 (0.46, 1.20) | | 0.80 (0.47, 1.35) |  |
| 12-17 | 1.18 (0.87, 1.59) | 1.09 (0.79, 1.51) | 0.97 (0.65, 1.45) | | 0.94 (0.61, 1.45) | 1.05 (0.68, 1.64) | | 1.08 (0.67, 1.73) |  |
| 18-23 | 1.00 (Reference) | 1.00 (Reference) | 1.00 (Reference) | | 1.00 (Reference) | 1.00 (Reference) | | 1.00 (Reference) |  |
| 24-29 | 1.16 (0.79, 1.72) | 1.01 (0.66, 1.54) | 1.05 (0.60, 1.85) | | 1.01 (0.54, 1.87) | 1.25 (0.67, 2.32) | | 1.23 (0.63, 2.39) |  |
| 30-35 | 1.10 (0.68, 1.79) | 0.98 (0.58, 1.66) | 1.15 (0.62, 2.16) | | 1.03 (0.51, 2.10) | 1.02 (0.50, 2.09) | | 0.85 (0.39, 1.86) |  |
| ≥ 36 | 1.10 (0.69, 1.76) | 1.09 (0.65, 1.81) | 0.73 (0.38, 1.40) | | 0.78 (0.38, 1.60) | 0.77 (0.37, 1.61) | | 0.82 (0.38, 1.78) |  |
| **SGA** |  |  |  | |  |  | |  |  |
| 0-5 | 0.95 (0.60, 1.51) | 0.92 (0.56, 1.52) | 0.67 (0.37, 1.21) | | 0.65 (0.34, 1.22) | 0.51 (0.25, 1.05) | | 0.55 (0.25, 1.21) |  |
| 6-11 | 1.18 (0.83, 1.68) | 1.20 (0.83, 1.74) | 0.98 (0.63, 1.53) | | 0.95 (0.59, 1.52) | 0.95 (0.56, 1.62) | | 0.95 (0.54, 1.66) |  |
| 12-17 | 0.95 (0.70, 1.31) | 1.03 (0.74, 1.43) | 0.79 (0.53, 1.18) | | 0.81 (0.53, 1.22) | 0.78 (0.49, 1.23) | | 0.78 (0.48, 1.25) |  |
| 18-23 | 1.00 (Reference) | 1.00 (Reference) | 1.00 (Reference) | | 1.00 (Reference) | 1.00 (Reference) | | 1.00 (Reference) |  |
| 24-29 | 0.87 (0.58, 1.31) | 0.84 (0.56, 1.28) | 0.72 (0.41, 1.26) | | 0.72 (0.40, 1.29) | 0.59 (0.30, 1.16) | | 0.61 (0.30, 1.23) |  |
| 30-35 | 1.59 (0.97, 2.60) | **1.75 (1.04, 2.97)** | 1.04 (0.55, 1.99) | | 1.10 (0.56, 2.17) | 0.89 (0.42, 1.85) | | 1.03 (0.47, 2.24) |  |
| ≥ 36 | 1.21 (0.68, 2.13) | 1.17 (0.64, 2.13) | 0.89 (0.40, 1.96) | | 1.02 (0.44, 2.39) | 0.79 (0.33, 1.90) | | 0.88 (0.34, 2.28) |  |
| **LBW^d^** |  |  |  | |  |  | |  |  |
| 0-5 | 1.64 (0.91, 2.97) | 1.68 (0.87, 3.23) | 1.39 (0.68, 2.86) | | 1.21 (0.54, 2.72) | 1.69 (0.74, 3.86) | | 1.35 (0.54, 3.40) |  |
| 6-11 | 1.37 (0.84, 2.21) | 1.44 (0.84, 2.45) | 1.09 (0.58, 2.05) | | 1.03 (0.51, 2.09) | 1.41 (0.70, 2.84) | | 1.31 (0.61, 2.81) |  |
| 12-17 | 1.19 (0.76, 1.86) | 1.16 (0.72, 1.88) | 1.00 (0.57, 1.77) | | 1.00 (0.54, 1.84) | 1.34 (0.71, 2.53) | | 1.29 (0.66, 2.54) |  |
| 18-23 | 1.00 (Reference) | 1.00 (Reference) | 1.00 (Reference) | | 1.00 (Reference) | 1.00 (Reference) | | 1.00 (Reference) |  |
| 24-29 | **1.65 (0.98, 2.79)** | 1.57 (0.90, 2.74) | 1.36 (0.68, 2.73) | | 1.46 (0.69, 3.10) | 1.70 (0.78, 3.75) | | 1.67 (0.71, 3.92) |  |
| 30-35 | **2.04 (1.04, 3.99)** | 1.91 (0.92, 3.95) | 1.31 (0.55, 3.11) | | 1.22 (0.46, 3.27) | 1.08 (0.41, 2.89) | | 1.05 (0.35, 3.13) |  |
| ≥ 36 | 1.80 (0.97, 3.32) | 1.85 (0.94, 3.64) | 1.33 (0.61, 2.91) | | 1.65 (0.68, 3.98) | 1.71 (0.71, 4.12) | | 1.89 (0.72, 4.93) |  |
| **NICU** |  |  |  | |  |  | |  |  |
| 0-5 | 0.94 (0.65, 1.38) | 0.93 (0.61, 1.41) | 1.14 (0.70, 1.85) | | 1.03 (0.60, 1.76) | 1.36 (0.79, 2.35) | | 1.26 (0.69, 2.32) |  |
| 6-11 | 0.95 (0.72, 1.27) | 1.01 (0.74, 1.37) | 0.93 (0.64, 1.35) | | 0.91 (0.60, 1.37) | 1.03 (0.69, 1.53) | | 0.95 (0.61, 1.48) |  |
| 12-17 | 1.15 (0.88, 1.52) | 1.24 (0.92, 1.67) | 1.14 (0.80, 1.64) | | 1.18 (0.79, 1.74) | 1.12 (0.76, 1.66) | | 1.13 (0.74, 1.74) |  |
| 18-23 | 1.00 (Reference) | 1.00 (Reference) | 1.00 (Reference) | | 1.00 (Reference) | 1.00 (Reference) | | 1.00 (Reference) |  |
| 24-29 | 0.99 (0.73, 1.35) | 0.97 (0.70, 1.35) | 0.90 (0.59, 1.38) | | 0.84 (0.54, 1.33) | 0.98 (0.61, 1.58) | | 0.96 (0.57, 1.59) |  |
| 30-35 | 1.12 (0.76, 1.65) | 1.02 (0.67, 1.54) | 1.18 (0.70, 1.97) | | 1.01 (0.57, 1.79) | 1.41 (0.78, 2.54) | | 1.14 (0.59, 2.19) |  |
| ≥ 36 | 1.44 (0.93, 2.24) | 1.37 (0.86, 2.19) | 1.21 (0.61, 2.39) | | 1.03 (0.50, 2.12) | 1.51 (0.71, 3.20) | | 1.34 (0.60, 2.97) |  |

IPI, interpregnancy interval; PTB, preterm birth; SGA, small for gestational age; LBW, low birthweight; NICU, neonatal intensive care unit; OR, odds ratio; CI, confidence interval

Bold indicates statistical significance at the 5% level

^a^ sample size for Scenario C varies depending on examined outcome: n=4,524 for PTB; n=4,430 for SGA; n=4,624 for LBW and n = 4,389 for NICU admission

^b^ adjusted model accounted for characteristics that varied or could potentially vary between pregnancies within the same individual: individual’s age, marital status, health insurance, prepregnancy BMI, chronic conditions, smoking during pregnancy, mode of delivery; pregnancy order included in the adjusted model to allow for different intercepts at the 2^nd^ and 3^rd^ births

Individual’s chronic conditions include having at least one of: heart disease, chronic hypertension, diabetes mellitus, thyroid disorders, asthma, renal diseases

^c^ due to small counts in some categories of variables, PTB scenario C is adjusted for the same variables, except maternal age and smoking during pregnancy

^d^ due to small counts in some categories of variables, LBW scenario B is adjusted for the same variables, except smoking and

LBW scenario C is adjusted for the same variables, except maternal age and smoking

**eTable 2.** Comparison of maternal and newborn characteristics at the second birth for individuals with ≥3 births (n=10,647) and individuals with only 2 births (n=38,592), NICHD consecutive pregnancies study, Utah, 2002-2010

|  | **Individuals with ≥ 3 birth** | **Individuals with 2 births^a^** |
| --- | --- | --- |
| **Total** | 10,647 | 38,592 |
| **Maternal characteristics** |  |  |
| IPI, months, mean (SD) | 17.1 (8.8) | 22.4 (13.0) |
| IPI, months |  |  |
| 0-5 | 715 (6.7) | 1,804 (4.7) |
| 6-11 | 2,247 (21.1) | 5,728 (14.8) |
| 12-17 | 3,157 (29.7) | 8,528 (22.1) |
| 18-23 | 2,343 (22.0) | 7,759 (20.1) |
| 24-29 | 1,223 (11.5) | 5,596 (14.5) |
| 30-35 | 561 (5.3) | 3,425 (8.9) |
| ≥ 36 | 401 (3.8) | 5,752 (14.9) |
| Race |  |  |
| White | 9,409 (88.5) | 33,039 (85.7) |
| Non-white | 1,226 (11.5) | 5,517 (14.3) |
| Age at birth, years, mean (SD) | 26.8 (4.0) | 28.4 (4.8) |
| Age at birth, years |  |  |
| 14-19 | 279 (2.6) | 710 (1.8) |
| 20-24 | 2,827 (26.6) | 7,627 (19.8) |
| 25-29 | 4,998 (46.9) | 15,501 (40.2) |
| 30-34 | 2,130 (20.0) | 10,497 (27.2) |
| ≥ 35 | 413 (3.9) | 4,257 (11.0) |
| Insurance |  |  |
| Private | 7,976 (74.9) | 28,297 (73.3) |
| Public | 2,671 (25.1) | 10,295 (26.7) |
| Marital status |  |  |
| Not married | 853 (8.1) | 3,980 (10.3) |
| Married | 9,792 (92.0) | 34,609 (89.7) |
| Prepregnancy BMI, kg/m^2^ |  |  |
| Underweight (<18.5) | 597 (5.7) | 1,679 (4.4) |
| Normal (18.5-25) | 6,107 (58.3) | 20,586 (54.1) |
| Overweight (25-30) | 2,278 (21.7) | 8,980 (23.6) |
| Obese (≥30) | 1,500 (14.3) | 6,778 (17.8) |
| Parity |  |  |
| 1 | 6,154 (57.8) | 20,600 (53.4) |
| 2 | 2,643 (24.8) | 8,625 (22.4) |
| ≥ 3 | 1,850 (17.4) | 9,367 (24.3) |
| Smoking during pregnancy |  |  |
| No | 10,387 (97.7) | 37,240 (96.6) |
| Yes | 247 (2.3) | 1,318 (3.4) |
| Alcohol use during pregnancy |  |  |
| No | 10,542 (99.2) | 37,807 (98.2) |
| Yes | 85 (0.8) | 677 (1.8) |
| Pre-existing diabetes mellitus | 175 (1.6) | 796 (2.1) |
| Pre-existing hypertension | 71 (0.7) | 496 (1.3) |
| Chronic conditions^b^ | 1,632 (15.3) | 6,748 (17.5) |
| Cesarean section | 1,459 (13.7) | 7,660 (19.9) |
| **Newborn characteristics** |  |  |
| Female | 5,069 (47.6) | 18,711 (48.5) |
| Birth weight, grams, mean (SD) | 3,361.0 (484.8) | 3,342.5 (488.3) |
| PTB | 737 (6.9) | 2,871 (7.4) |
| SGA | 557 (5.2) | 2,296 (6.0) |
| LBW | 367 (3.5) | 1,542 (4.0) |
| NICU admission | 724 (6.8) | 3,169 (8.2) |

Percentages are column percentages.

IPI, interpregnancy interval; SD, standard deviation; BMI, body mass index; PTB, preterm birth; LBW, low birth weight; SGA, small for gestational age; NICU, neonatal intensive care unit.

^a^ For individuals with only 2 births, the same inclusion/exclusion criteria were applied: both singleton and both liveborn births.

^b^ Individual’s chronic conditions include having at least one of the following conditions: heart disease, chronic hypertension, diabetes mellitus, thyroid disorder, asthma, renal disease.

Missing for individuals with ≥ 3 births: marital status (n=2), BMI (n=165), maternal smoking (n=13), alcohol use during pregnancy (n=20), birth weight (n=7), SGA (n=14), LBW (n=7) at 2^nd^ birth.

Missing for individuals with only 2 births: race (n=36), BMI (n=569), smoking (n=34), alcohol (n=108); birth weight (n=10), SGA (n=18), LBW (n=10) at 2^nd^ birth.

**eTable 3.** Number (%) of PTB, SGA, and LBW events by birth order and odds ratios (ORs) and 95% confidence intervals (CI) for the association between interpregnancy interval and adverse perinatal outcomes in individuals with only 2 liveborn singleton pregnancies (between-individual comparison, n=38,592), NICHD consecutive pregnancies study, Utah, 2002-2010

| **IPI, months** | **n (%) of events at 2^nd^ birth** | **Unadjusted (model 1)** | **Adjusted ^a^ (model 2)** | **Adjusted ^b^ (model 3)** |
| --- | --- | --- | --- | --- |
| **PTB** | 2,871 (7.4) |  |  |  |
| 0-5 | 252 (14.0) | **2.26 (1.92, 2.65)** | **1.99 (1.68, 2.35)** | **1.86 (****1.56, 2.22)** |
| 6-11 | 462 (8.1) | **1.22 (1.07, 1.39)** | **1.16 (1.02, 1.33)** | 1.13 (0.98, 1.30) |
| 12-17 | 597 (7.0) | 1.05 (0.93, 1.18) | 1.03 (0.91, 1.17) | 1.05 (0.93, 1.20) |
| 18-23 | 521 (6.7) | 1.00 (Reference) | 1.00 (Reference) | 1.00 (Reference) |
| 24-29 | 361 (6.5) | 0.96 (0.83, 1.10) | 0.95 (0.83, 1.10) | 0.95 (0.82, 1.10) |
| 30-35 | 241 (7.0) | 1.05 (0.90, 1.23) | 1.02 (0.86, 1.19) | 0.98 (0.83, 1.15) |
| ≥ 36 | 437 (7.6) | 1.14 (1.00, 1.30) | 1.08 (0.94, 1.24) | 1.03 (0.90, 1.19) |
| **SGA** | 2,296 (6.0) |  |  |  |
| 0-5 | 139 (7.7) | **1.40 (1.15, 1.71)** | 1.11 (0.90, 1.37) | 1.05 (0.85, 1.30) |
| 6-11 | 350 (6.1) | 1.09 (0.95, 1.26) | 1.02 (0.88, 1.18) | 0.97 (0.83, 1.13) |
| 12-17 | 483 (5.7) | 1.01 (0.88, 1.15) | 1.00 (0.87, 1.14) | 0.99 (0.86, 1.14) |
| 18-23 | 436 (5.6) | 1.00 (Reference) | 1.00 (Reference) | 1.00 (Reference) |
| 24-29 | 310 (5.6) | 0.99 (0.85, 1.15) | 1.00 (0.86, 1.16) | 0.99 (0.85, 1.15) |
| 30-35 | 199 (5.8) | 1.04 (0.87, 1.23) | 1.04 (0.88, 1.24) | 1.04 (0.87, 1.24) |
| ≥ 36 | 379 (6.6) | **1.19 (1.03, 1.37)** | **1.15 (1.00, 1.34)** | 1.14 (0.98, 1.32) |
| **LBW** | 1,542 (4.0) |  |  |  |
| 0-5 | 132 (7.3) | **2.33 (1.88, 2.90)** | **1.85 (1.47, 2.33)** | **1.67 (****1.32, 2.11)** |
| 6-11 | 261 (4.6) | **1.41 (1.18, 1.68)** | **1.28 (1.07, 1.53)** | **1.22 (****1.01, 1.47)** |
| 12-17 | 292 (3.4) | 1.05 (0.88, 1.24) | 1.04 (0.87, 1.23) | 1.05 (0.88, 1.25) |
| 18-23 | 254 (3.3) | 1.00 (Reference) | 1.00 (Reference) | 1.00 (Reference) |
| 24-29 | 186 (3.3) | 1.02 (0.84, 1.23) | 1.02 (0.84, 1.24) | 1.02 (0.84, 1.24) |
| 30-35 | 133 (3.9) | 1.19 (0.96, 1.48) | 1.17 (0.94, 1.46) | 1.14 (0.92, 1.43) |
| ≥ 36 | 284 (4.9) | **1.54 (1.29, 1.82)** | **1.45 (1.21, 1.73)** | **1.40 (1.17, 1.68)** |
| **NICU** | 3,169 (8.2) |  |  |  |
| 0-5 | 217 (12.0) | **1.63 (1.38, 1.92)** | **1.50 (1.26, 1.78)** | **1.43 (****1.20, 1.70)** |
| 6-11 | 477 (8.3) | 1.08 (0.95, 1.22) | 1.03 (0.91, 1.17) | 1.01 (0.89, 1.15) |
| 12-17 | 633 (7.4) | 0.95 (0.85, 1.07) | 0.93 (0.83, 1.05) | 0.94 (0.84, 1.06) |
| 18-23 | 602 (7.8) | 1.00 (Reference) | 1.00 (Reference) | 1.00 (Reference) |
| 24-29 | 444 (7.9) | 1.03 (0.90, 1.16) | 1.01 (0.89, 1.15) | 1.01 (0.88, 1.15) |
| 30-35 | 274 (8.0) | 1.03 (0.89, 1.20) | 1.03 (0.88, 1.19) | 1.00 (0.86, 1.17) |
| ≥ 36 | 522 (9.1) | **1.19 (1.05, 1.34)** | **1.15 (1.01, 1.30)** | 1.12 (0.99, 1.27) |

IPI, interpregnancy interval; PTB, preterm birth; LBW, low birth weight; SGA, small for gestational age; NICU, neonatal intensive care unit.

Bold indicates statistical significance at the 5% level.

**^a^** adjusted model accounted for parity, individual’s race, individual’s age, marital status, health insurance, prepregnancy BMI, maternal chronic conditions, maternal smoking at the first pregnancy.

^b^ models for PTB, SGA and LBW in addition, adjusted for the outcome of the first birth (SGA and PTB); model for NICU admission in addition, adjusted for the outcome of the first birth (SGA and PTB, NICU admission).

**eTable 4**. Odds Ratios (ORs) and 95% confidence intervals (CI) for the association between post-birth interval (between 2^nd^ and 3^rd^ births) and adverse perinatal outcomes at the 2^nd^ birth for individuals with ≥3 singleton live births (n = 10,647), NICHD consecutive pregnancies study, Utah, 2002-2010

| **IPI, months** | **Unadjusted OR (95% CI)** | **Adjusted^a^ OR (95% CI)** |
| --- | --- | --- |
| **PTB** |  |  |
| 0-5 | **2.69 (2.00, 3.67)** | **2.00 (1.41, 2.83)** |
| 6-11 | **1.46 (1.14, 1.87)** | **1.35 (1.04, 1.76)** |
| 12-17 | 1.19 (0.95, 1.50) | 1.18 (0.92, 1.50) |
| 18-23 | 1.00 (Reference) | 1.00 (Reference) |
| 24-29 | 1.09 (0.83, 1.41) | 1.13 (0.86, 1.50) |
| 30-35 | 1.15 (0.84, 1.59) | 1.16 (0.83, 1.62) |
| ≥ 36 | **1.40 (1.00, 1.94)** | 1.26 (0.89, 1.78) |
| **SGA** |  |  |
| 0-5 | **1.99 (1.39, 2.85)** | **1.62 (1.09, 2.42)** |
| 6-11 | 1.05 (0.79, 1.40) | 1.08 (0.80, 1.46) |
| 12-17 | 1.16 (0.91, 1.49) | 1.20 (0.93, 1.55) |
| 18-23 | 1.00 (Reference) | 1.00 (Reference) |
| 24-29 | 1.07 (0.81, 1.43) | 1.10 (0.82, 1.48) |
| 30-35 | 0.93 (0.64, 1.34) | 0.91 (0.62, 1.33) |
| ≥ 36 | 0.77 (0.50, 1.19) | 0.80 (0.52, 1.24) |
| **LBW** |  |  |
| 0-5 | **3.42 (2.29, 5.10)** | **2.52 (1.61, 3.92)** |
| 6-11 | 1.38 (0.97, 1.97) | 1.28 (0.88, 1.86) |
| 12-17 | **1.42 (1.04, 1.95)** | 1.39 (0.99, 1.94) |
| 18-23 | 1.00 (Reference) | 1.00 (Reference) |
| 24-29 | 1.09 (0.75, 1.59) | 1.16 (0.79, 1.71) |
| 30-35 | 0.84 (0.50, 1.40) | 0.82 (0.49, 1.38) |
| ≥ 36 | 1.26 (0.78, 2.04) | 1.12 (0.68, 1.86) |
| **NICU admission** |  |  |
| 0-5 | **1.64 (1.18, 2.29)** | 1.28 (0.89, 1.84) |
| 6-11 | 1.09 (0.85, 1.40) | 1.04 (0.80, 1.34) |
| 12-17 | 0.95 (0.76, 1.19) | 0.93 (0.74, 1.17) |
| 18-23 | 1.00 (Reference) | 1.00 (Reference) |
| 24-29 | 1.03 (0.80, 1.32) | 1.06 (0.82, 1.37) |
| 30-35 | 1.05 (0.77, 1.42) | 1.04 (0.76, 1.43) |
| ≥ 36 | 0.93 (0.66, 1.31) | 0.90 (0.63, 1.28) |

IPI, interpregnancy interval; PTB, preterm birth; LBW, low birth weight; SGA, small for gestational age; NICU, neonatal intensive care unit.

Modeling perinatal outcomes of second born infant using post-birth intervals (interval between 2^nd^ and 3^rd^ birth).

Bold indicates statistical significance at the 5% level.

^a^ Adjusted model for PTB, SGA and LBW accounted for parity, individual’s race, individual’s age, marital status, health insurance, prepregnancy BMI, maternal chronic conditions, maternal smoking at the second pregnancy, PTB and SGA at the first pregnancy; adjusted model for NICU admission, in addition, adjusted for the NICU admission at the first pregnancy.
